# Multicolor fluorescence microscopy using static light sheets and a single-channel detection

**DOI:** 10.1117/1.JBO.24.1.016501

**Published:** 2019-01-05

**Authors:** Jacob Licea-Rodriguez, Alfredo Figueroa-Melendez, Konstantinos Falaggis, Marcos Plata-Sanchez, Meritxell Riquelme, Israel Rocha-Mendoza

**Affiliations:** a Centro de Investigación Científica y de Educación Superior de Ensenada, Department of Optics, Ensenada, Baja California, Mexico; b Cátedras Conacyt, Centro de Investigación Científica y de Educación Superior de Ensenada, Ensenada, Baja California, Mexico; c Centro de Investigación Científica y de Educación Superior de Ensenada, Department of Microbiology, Ensenada, Baja California, Mexico; d University of North Carolina, Department of Mechanical Engineering and Engineering Science, Charlotte, North Carolina, United States

**Keywords:** medical and biological imaging, fluorescence microscopy, multiple imaging, laser beam combining

## Abstract

We present a multicolor fluorescence microscope system, under a selective plane illumination microscopy (SPIM) configuration, using three continuous wave-lasers and a single-channel-detection camera. The laser intensities are modulated with three time-delayed pulse trains that operate synchronously at one third of the camera frame rate, allowing a sequential excitation and an image acquisition of up to three different biomarkers. The feasibility of this imaging acquisition mode is demonstrated by acquiring single-plane multicolor images of living hyphae of *Neurospora crassa.* This allows visualizing simultaneously the localization and dynamics of different cellular components involved in apical growth in living hyphae. The configuration presented represents a noncommercial, cost-effective alternative microscopy system for the rapid and simultaneous acquisition of multifluorescent images and can be potentially useful for three-dimensional imaging of large biological samples.

## 1 Introduction

Developmental biology studies require both two-dimensional (2-D) and three-dimensional (3-D) visualization of different dynamic microstructures, marked with specific fluorescent proteins, to elucidate their functionality in a determined biological process. For those purposes, confocal laser scanning microscopy (CLSM) and/or multiphoton microscopy (MPM) have been successfully used to excite multiple fluorescent markers and visualize selectively different planes of the samples (obtaining the so-called optically sectioned images).^1^^–^^5^ However, these techniques are not suited to visualize large volumetric samples because the image is constructed sequentially point by point while the laser is raster scanning the sample, imposing a strong limit on the acquisition speed. Other systems, such as spinning disk confocal systems (SDCS), use a rotating disk with a pattern of pinholes installed in a microscope plane that is conjugated with the specimen.^6^ SDCS provide faster image acquisition, and therefore, are better suited to track the dynamics of microstructures that move within the scanning frame rate of a CLSM, producing images without jagged edges and better definition. However, since in SDCS the disk pinhole openings are commonly wide for better signal collection, this technique does not allow an optical sectioning with the same thinness as those attainable by CLSM or MPM. In either case, 3-D imaging using CLSM, MPM, or SDCS, is not suited for large biological specimens due to the objective high numerical aperture and short working distance commonly utilized in those techniques.

Light-sheet fluorescence microscopy (LSFM), based on a planar illumination of the sample, has revolutionized in the last decade optical 3-D imaging of biological specimens.^7^^–^^10^ LSFM techniques are made possible by decoupling the light excitation and detection optical paths. These planar illumination strategies allow achieving wide-field imaging while minimizing fluorescence from out-of-focus. In addition, they provide faster image acquisition and more efficient signal detection using high-efficiency cameras. According to the way the plane of light is formed, two main configurations are commonly used in LSFM: the so-called selective plane illumination microscopy (SPIM) and the digital scanned laser microscopy.

In the work presented here, an SPIM configuration is used. In this configuration, the sheet of light formed at the focus of a cylindrical lens is employed to illuminate a plane in the sample. The generated fluorescence signal is collected by an objective lens, with the optical axis orthogonal to the illumination plane that projects the fluorescence image onto a camera. SPIM has been used to visualize large living biological systems such as zebrafish,^11^^,^^12^ *Drosophila melanogaster* embryos,^13^ *Caenorhabditis elegans*,^14^ tumor cell spheroids,^15^^,^^16^ and *Arabidopsis thaliana*.^17^

Different LSFM configurations have been proposed to perform multicolor 3-D imaging.^18^^–^^21^ For instance, Krieger et al. performed dual color fluorescence imaging using a single camera and two separate color channels, while Jahr et al. used a diffractive unit to spectrally split the images onto a camera in order to obtain hyperspectral images. Additionally, Mahou et al. implemented a two-photon multicolor light-sheet microscope using a femtosecond laser and an optical parametric oscillator to obtain multicolor two-photon excitation using a single camera. For that, the spectral channels were spatially split and projected onto the camera using an image splitter. However, the use of pulsed excitation sources increases the costs of multicolor light-sheet-based microscopy systems. In all former cases, fine alignments and considerable image processing efforts are typically needed to overlap the images correctly. Other multicolor SPIM setups require two or more detection cameras,^22^^–^^27^ but the main aim of these arrangements was to improve the image by reducing or compensating scattered light and aberrations effects that normally occur on single-color images and, to the best of our knowledge, were not utilized for multicolor imaging purposes.

Recently, Girstmair et al.^28^^,^^29^ demonstrated the benefits of implementing two color dual-sided SPIM imaging using an open access platform and concluded that SPIM can be in principle accessible to anyone interested in having their own light-sheet microscope, but recognized that a significant investment of time and money is required. The aim of this work was to obtain a noncommercial cost-effective microscope system to perform multifluorescent (three color) SPIM imaging for the fast acquisition of up to three different biomarkers, using three synchronized continuous wave (cw) lasers and a single-camera detection. The feasibility of tracking fast biological processes is demonstrated by imaging living cells of the filamentous fungus *N. crassa* expressing two different fluorescent proteins and stained with a fluorescent dye. The system was assembled under a SPIM configuration for its potential use to perform 3-D biological studies on large samples.

## 2 Methods

### 2.1 Sample Preparation

#### 2.1.1 Fluorescein/rhodamine and fluorescent beads

For the light-sheet characterization, a mixed solution containing fluorescein and rhodamine was prepared. First, fluorescein and rhodamine were dissolved separately in distilled water to a concentration of 0.5 mM. Each solution was kept separately into an Eppendorf tube. A quartz cuvette (Hellma Analytics, 100-QS) was filled with a 1:30 rhodamine:fluorescein mixture. The fluorescence emission of fluorescein was used to characterize the light sheets excited with the 445- and 488-nm laser wavelengths, whereas the fluorescence emission of rhodamine was used to characterize the light sheet excited with the 561-nm laser wavelength.

To measure the spatial resolution of the system, a sample containing fluorescent microbeads of 0.16  μm (Dye XC, concentration 1%, Estapor Microspheres) immersed in agarose was employed. The excitation wavelength range of these microbeads is from 440 to 520 nm, with three maxima at 470, 480, and 490 nm. The fluorescence emission ranges from 500 to 600 nm, with two maxima at 525 and 560 nm.^30^ A 1:10 bead–water solution was prepared and mixed with melted 1.5% agarose at 1:100 and a 100-μL drop of the resulting bead–agar sample was cooled down at room temperature during 5 min on a coverslip until solidification. A cube of the solidified sample was cut and mounted on the SPIM system holder facing toward the collection objective.

#### 2.1.2 Biological samples

**Fig. 1:**
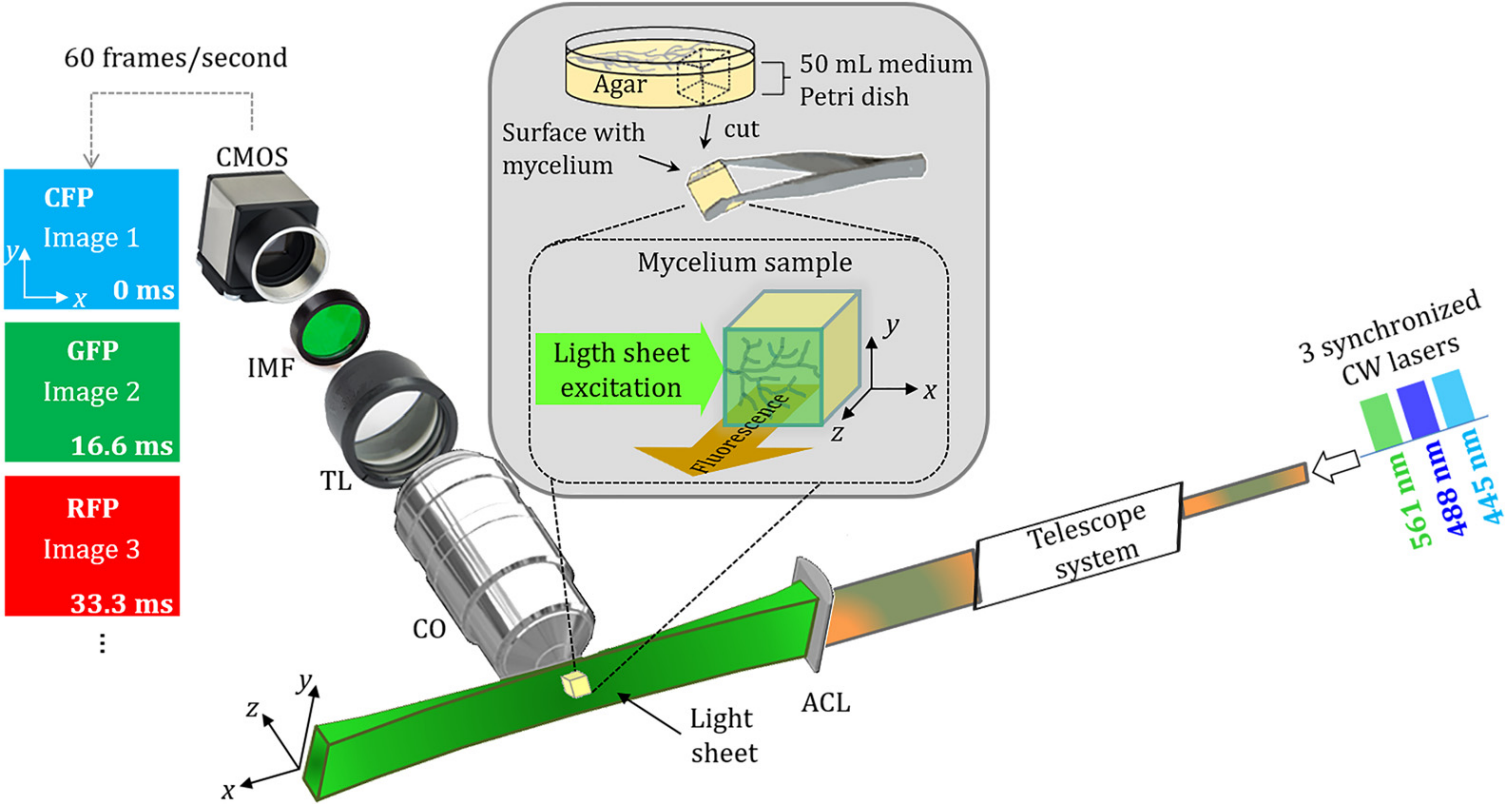
Multicolor imaging setup based on an SPIM configuration. ACL, achromatic cylindrical lens; CO, collection objective; IMF, interferometric multiband filter; TL, tube lens; CMOS, camera; and xyz, laboratory coordinate system. Inset: cutting and mounting geometry of a cube of agar containing the growing mycelium sample. Dashed square: excitation–collection geometry of the cube containing the sample. The leftmost colored boxes represent the image sequence acquisition at the maximum frame acquisition rate of the camera (60 fps), which leads to have 20 fps for each color that corresponds to an exposure time of 16.6 ms.

To test the feasibility of the system, a mix of same mating type conidia from different *Neurospora crassa* strains ($10^{6}\text{ conidia }{\mathrm{mL}}^{-1}$ each) was inoculated in Petri dishes containing 25 mL of Vogel’s minimal medium (VMM)^31^ solidified with 1.5% agar (AGARMEX, S.A de C.V) and incubated overnight at 30°C. Strains expressing H1-RFP (RFP-tagged histone 1 as a nuclear marker) and BML-GFP (green fluorescent protein-tagged β-tubulin as a microtubular marker) were used. The strain expressing GFP-tagged microtubules was obtained by Michael Freitag and has been previously published as FGSC# N2526 (Freitag et al.;^32^ ridRIP4; $his\text{-}3^{+}∷Pccg\text{-}1\text{-}Bml^{+}\text{-}{\mathrm{sqfp}}^{+}$; *Mat A*). Strain NMF138 ($\Delta mus51∷{\mathrm{bar}}^{+}$; $his\text{-}3^{+}∷Pccg\text{-}1∷h1∷rfp$; *Mat A*) expressing H1-RFP was also obtained by M. Freitag’s lab. During the incubation time, the mycelia of these strains were fused due to self–self recognition, while allowing co-expression of genotypically distinct nuclei. To fluorescently stain cell walls, a 0.1%  w/v solophenyl flavine 7GFE stock solution was prepared, diluted 10-fold in liquid VMM (100  μg/mL) and applied directly to the mycelium using a pipette. A $1.5\text{-}{\mathrm{cm}}^{2}$ block of the agar medium containing the edge of the mycelium was cut out using a single-edged razor blade and mounted carefully with flat tip tweezers (see inset of Fig. 1) on the system holder with the mycelium facing toward the collection objective lens.

### 2.2 Experiment

#### 2.2.1 Multicolor light-sheet fluorescence microscope setup

The layout to perform multicolor light-sheet imaging based on an SPIM configuration is shown in Fig. 1. The system utilizes a triple sequential pulse excitation by modulating the intensity of three cw-lasers at different wavelengths (explained in Sec. 2.2.2), which are recombined by two dichroic filters (DFs) (Thorlabs, MD499 and MD480). The recombined beams are expanded and collimated via a telescope system formed by an 8-mm focal length aspheric lens and a 50-mm focal length collimator lens. The resulting collimated beams are around 8-mm in diameter beams [measured at the full-width half-maximum (FWHM) value]. The light sheets are generated by an achromatic cylindrical lens (ACL) (Thorlabs ACY254, 50-mm focal length) and overlapped in the xy sample plane. The fluorescence signals generated at the illuminated plane are collected along the z direction by an infinite corrected long working distance objective lens CO (Mitutoyo, 50×; NA: 0.55; WD: 13 mm) placed at 90 deg to the sample plane. A tube lens (TL) [Thorlabs, Transistor–Transistor Logic (TTL200); 200-mm focal length] is used to form the image of the fluorescent structures onto a high-sensitive (CMOS) camera (Thorlabs, DCC3240N), with average quantum efficiency of 65% in the range of 450 to 700 nm, and a maximum frame acquisition rate of 60 images per second. To filter out the lasers excitation and achieve an efficient signal collection for each excited fluorophore, a high-quality interferometric multiband filter (IMF) (Semrock, Em01-R488/568-25) is employed. The sample is mounted onto a custom-designed holder attached to a computer-controlled xyz linear translational stage (Thorlabs, NanoMax 300).

#### 2.2.2 Lasers modulation and CMOS synchronization

**Fig. 2:**
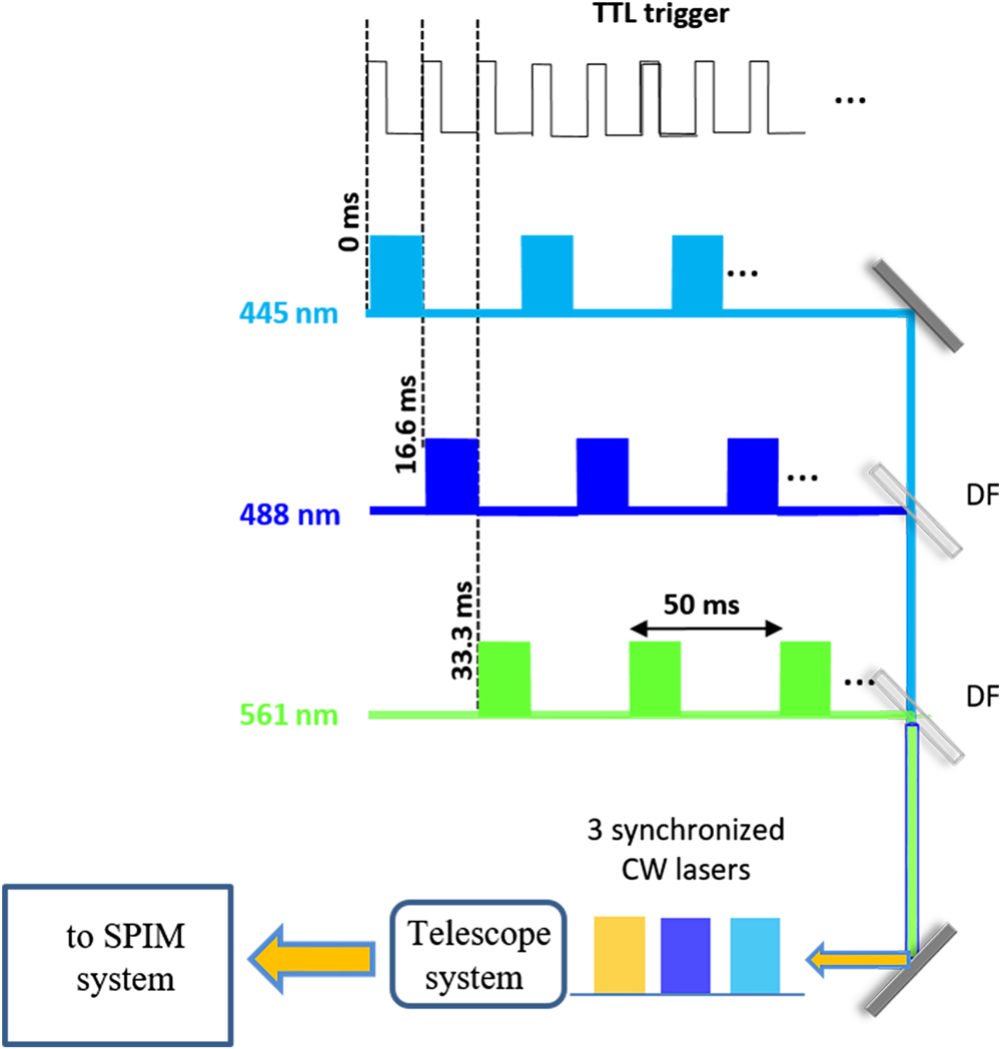
Intensity modulation and synchronization of the three lasers using four different square wave signals generated via an Arduino board. The laser beams are recombined by two DFs, then expanded and collimated by a telescope system, and finally delivered to the SPIM system shown in Fig. 1.

The intensity modulation and synchronization of the three lasers (Coherent, Obis; operating at 445, 488, and 561 nm) was performed via four different square wave signals generated via an Arduino USB board (Arduino, UNO). The first corresponded to a TTL signal used as the master signal and to continuous trigger the CMOS camera at a frequency $f_{\mathrm{TTL}}$. The other three signals were at $1/3f_{\mathrm{TTL}}$ and were time delayed one after the other (at T=0, 0, $1/f_{\mathrm{TTL}}$, $2/f_{\mathrm{TTL}}$ respectively) to obtain sequential light-sheet frame acquisition (see Fig. 2). Notably, the frequency $f_{\mathrm{TTL}}$ was adjusted to match the desired camera frame rate to a maximum achievable frame rate per light sheet of 20 frames per second. In practice, however, the camera frame rate was dictated by the exposure time needed to take each image. In our experiments, exposure time was set to 100 ms and the acquisition frame rate $f_{\mathrm{TTL}}=9$ images per second (3 frames per second for each laser). A similar approach using an Arduino board for the laser synchronization has been reported, where an open-source diode laser combiner and software sequence controller were used.^33^ Here MATLAB software is used to synchronize the lasers modulation and image acquisition via the Arduino board (based on Fig. 2 layout).

#### 2.2.3 Multiple fluorescent emission and single-channel detection

**Fig. 3:**
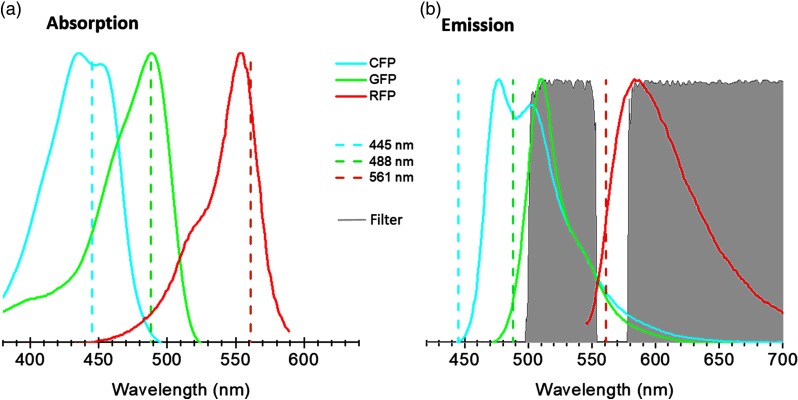
(a) Absorption and (b) emission spectra of CFP, GFP, and RFP fluorophores. The spectra were obtained from the database of fluorescent dyes.^34^ The excitation wavelengths are represented in dashed lines. The gray curve represents the transmission spectrum of the multiband filter used in the experiments (obtained from Ref. 35).

The laser wavelengths utilized in our system (445, 488, and 561 nm) allow the possibility of exciting many different fluorescent proteins triads. For instance, one can efficiently excite cyan fluorescent protein (CFP), GFP, and red fluorescent protein (RFP), respectively. This is shown in Fig. 3, where the absorption (a) and emission (b) spectra are shown, and the dashed lines indicate the used laser wavelengths. The wavelength of the blue lasers at 445 and 488 nm lays at the central part of the absorption spectra for the case of CFP and GFP, respectively; while the green laser (561 nm) lays in more than 80% of the normalized absorption of RFP. The modulation and time-delayed synchronization of the laser intensities, combined with a high blocking (OD>5.5) IMF (Semrock, Em01-R488/568-25) in the collection path [see filled gray curves in Fig. 3(b)], ensuring the individual fluorescent signal collection without simultaneous laser excitation as well as avoiding any bleed-through effects. Special care should be taken when using fluorophores with broad excitation/emission spectra, or with long Stokes shifts, as these can contribute to signaling crosstalk. In such case, additional specific bandpass emission filter needs to be used.

## 3 Results

### 3.1 Light-Sheets Characterization

**Fig. 4:**
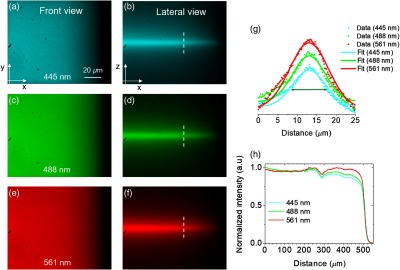
Light-sheets characterization. Front views using the excitation wavelengths at (a) 445 nm, (c) 488 nm, and (e) 561 nm. Lateral views for (b) 445 nm, (d) 488 nm, and (f) 561 nm. The intensity profiles along the x and z direction used to measure the effective FoV and the light-sheet thickness are presented in (h) and (g), respectively. (g) The intensity profiles corresponding to 488 and 561 nm were rescaled by a factor of 1.25 and 1.5, respectively, for better visualization. The green arrow indicates the FWHM value used to measure the thickness $w_{0}$ for the light sheet generated by the 445-nm laser. The intensity profiles in (h) were taken using the 10× collection objective.

To characterize the light sheets, a quartz cuvette was filled with the rhodamine–fluorescein mixture. The front views of the fluorescent light sheets are shown in Fig. 4 for the 445-nm [Fig. 4(a)], 488-nm [Fig. 4(c)], and 561-nm [Fig. 4(e)] excitation wavelengths. The beams incidence was from right to left. The total imaged area at the CMOS sensor was $120\times 100\;\mu m^{2}$ using a 50× (0.55 NA) collection objective. In the images, the right side edge of the cuvette is visualized. This edge was imaged on purpose so that the light-sheet beam waist $w_{0}$ of each beam was focused near to it. This can be better visualized in Figs. 4(b), 4(d), and 4(f), where the lateral views of the three light sheets are shown. As expected, slight differences in the beam waists’ dimensions and the focal positions are clearly visible due to the minimal chromatic aberrations of the employed ACL, where thinner light sheets and nearest focusing occur for shorter laser wavelengths. The dashed white lines indicate the beam waists’ locations and Fig. 4(g) shows the normalized intensity profiles along z axis. The measured thickness $w_{0}$ for each light sheet, using the FWHM criteria, was 9.1  μm for 445 nm, 9.6  μm for 488 nm, and 11.5  μm for 561 nm. The focal point positions of the 488-and 561-nm lasers are shifted with respect to the 445-nm laser by 50 and 95  μm, respectively. This was in accordance with the shifted focal points specified by the vendors (Thorlabs) of 60 and 100  μm.

Based on the definition for the Rayleigh range for Gaussian beams ($z_{R}=\pi w_{0}^{2}/\lambda$),^36^ the confocal parameter $b=2z_{R}$ is used to estimate the depth of focus (DoF) along the propagation direction (x axis) of the excitation light using the cylindrical lens. The computed DoFs for each wavelength were 1.169-, 1.186-, and 1.48-mm length for 455, 488, and 561 nm, respectively. Therefore, the expected overlapped area of the light sheets is considerably larger than the field–of-view (FoV) imaged with the utilized collection objective. To prove this experimentally, the intensity profiles along the x axis of the three different light sheets were characterized using a 10× (0.25 NA) collection objective (images not shown); with this objective, the total imaged area at the CMOS sensor was $560\times 450\;\mu m^{2}$. The resulting normalized intensity profiles are shown in Fig. 4(h). The intensity profiles are similar from 0  μm (where the beam waists are located) to 250  μm, then separate due to the ACL achromatic aberration, and finally decay at around 500  μm as a result of the edge of the quartz cuvette. The region where the intensity profile is similar extends up to 500  μm in length approximately and determines the zone where the multicolor images were analyzed. This length confirmed that the DoF of the three light sheets were in effect larger than the FoV of our multicolor light-sheet system ($∼120\times 100\;\mu m^{2}$) when using the 50× collection objective.

### 3.2 Lateral and Axial Resolution Estimation

In fluorescence microscopy, the image is formed by the convolution of the object and the FWHM of the point spread function (PSF) of the optical microscope system. The width of the PSF is the minimum resolvable unit of an optical microscope and is given by the Abbe diffraction limit formula $\delta_{\mathrm{FWHM}}=\lambda /(2\mathrm{NA})$, with λ as the fluorescence wavelength and NA is the numerical aperture of the objective lens. In practice, the PSF can be directly measured by taking the image of fluorescent beads smaller than $\delta_{\mathrm{FWHM}}$.^37^

The lateral and axial resolution of our SPIM system was estimated using the samples containing 0.16-μm fluorescent microbeads (see Sec. 2.1.1) with the laser wavelength at 488 nm for the excitation. The resulting resolutions were 0.8 and 4.5  μm, respectively. To measure that, 220 planes of the sample, separated 0.05  μm each, were imaged covering 11  μm of axial depth (the actual light-sheet width). Finally, the captured images were stored as a z-stack in TIFF format.

**Fig. 5:**
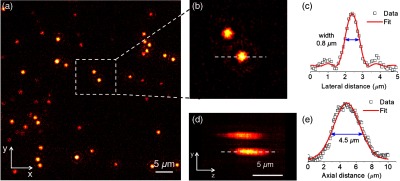
(a) Image of a single plane of fluorescent beads immersed in agar, (b) ROI image selected to measure the values of the PSFs, (c) lateral xy intensity profile of the bottom bead measured along the dashed line of (b), (d) ROI image in the zy direction, and (e) zy intensity profile measured along the dashed line of (d).

Figure 5(a) shows the image of a single plane of one z-stack, where different microbeads are focused within the collection objective DoF (∼0.9  μm according to the vendor specifications) and a selected region of interest (ROI), of around $10\times 10\;{\mu m}^{2}$, is indicated with a dashed square. The ROI is shown in Fig. 5(b) and the intensity profile of the PSF of one of the two microbeads is plotted in Fig. 5(c). The fitted ${\mathrm{sinc}}^{2}$ curve gives a value of ∼0.8  μm for the lateral resolution measured at the FWHM. Otherwise, Fig. 5(d) shows the zy plane of the selected ROI and the corresponding intensity profile of the PSF along z-direction is plotted in Fig. 5(e). The fitted Gaussian curve gives a value of ∼4.5  μm for the axial resolution measured at the FWHM.

### 3.3 N. crassa Imaging

The versatile imaging capability of this system is demonstrated for the case of growing hyphae of *N. crassa* expressing multiple fluorescent markers. Notice that, due to the typical hyphae dimensions and the light-sheet thicknesses achieved in our system, this biological system is not ideal for 3-D imaging. However, we decided to use it in order to demonstrate the rapid acquisition of multifluorescent images on a dynamic biological process. It is worth mentioning that other biological samples, such as clarified mouse brain and nonmelanized fungal fruiting bodies, would be ideal in the proposed imaging modality to perform 3-D multifluorescent imaging.

**Fig. 6:**
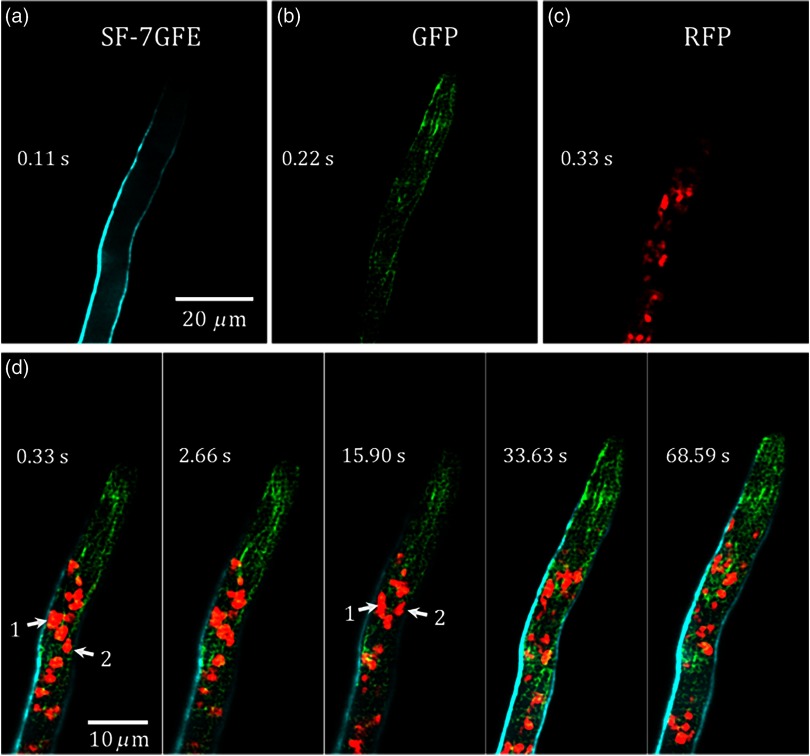
Multicolor imaging of a living hypha of *N. crassa*: (a) cell wall stained with solophenyl flavine 7GFE dye, (b) microtubules tagged with GFP, and (c) nuclei tagged with RFP. (d) Time lapsed merged images showing the dynamics of the different structures involved in hyphal growth.

**Fig. 7:**
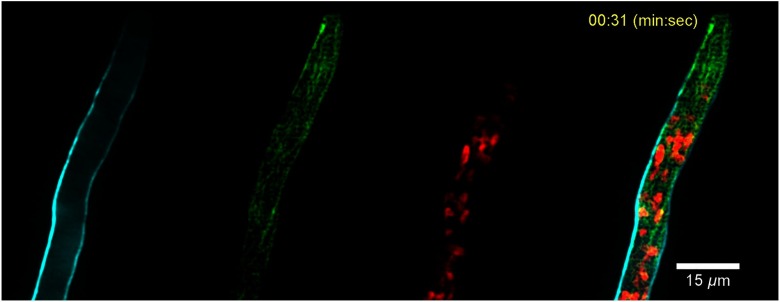
Multicolor imaging of a living hypha of *N. crassa* (Video 1, MPEG, 5.05 MB [URL: https://doi.org/10.1117/1.JBO.24.1.016501.1]).

Figure 6(a) shows the image of a single plane corresponding to the fluorescence emitted by the solophenyl flavine 7GFE (SF-7GFE) dye, which stains the cell wall. Figure 6(b) shows cytoplasmic microtubules, in which ß-tubulin subunits are tagged with the GFP, whereas Fig. 6(c) shows the nuclei, in which histone 1, H1 is tagged with the RFP. The excitation average power used was 1 mW for the 445-nm laser and 5 mW for 488- and 561-nm lasers. Finally, a time lapsed merged image is shown in Fig. 6(d), where all the stained structures are distinguished. Here five representative images at 0.33, 2.66, 15.9, 33.63, and 68.59 s were selected to depict hyphal growth. The dynamics of different structures involved in such growth can be observed in Fig. 7; where a refocusing of the sample is also appreciated. The measured velocity of nuclei 1 and 2 (indicated with arrows) is calculated to be 0.23 and 0.52  μm/s, respectively. It is worth mentioning that since the acquisition frame rate is 9 frames per second, each consecutive image is actually delayed 110 ms. However, considering the computed velocities, this would imply an average displacement of around 0.12  μm, which is below the resolution limit of our system, and therefore, is negligible. The acquisition frame rate required to obtain similar results using a commercial CLSM (Olympus FV1000) is about 10 times slower; every single merged image takes around 3 s in a sequential scanning (line by line) mode.

## 4 Discussion

The imaging configuration proposed here represents an alternative and cost-effective fluorescence microscope system capable of acquiring rapidly three wide field fluorescent images with good optical resolution. Since the system was performed under a light-sheet configuration, it is also capable of performing optical sectioning, which is useful for 3-D biological studies of the internal dynamics of large specimens like *C. elegans* or to study the internal structures of bulky samples like transgenic and clarified mouse brains.

Commercially available LSFM systems capable of developing multicolor imaging could be expensive depending on the system configuration, i.e., with single- or double-channel excitation/signal collection, and the number of laser sources. We set out to implement a multicolor fluorescence imaging system based on an SPIM configuration for its simplicity to couple three different static light sheets. The estimated cost of our system was around $60,000 USD, which included mainly the cost of the three laser sources, the xyz translation stage system, the CMOS camera, and the optics (ACL, filters, and objective).

In contrast, more expensive commercial systems incorporate more efficient CMOS cameras, specialized collection objectives (high NA and long working distance) for LSFM, and an immersion chamber for refractive index matching. Therefore, unavoidably the drawbacks in our system were: lower signal detection/collection efficiency and lower spatial resolution. This hindered the elucidation of submicron structures of our samples, and also the acquisition speed we could achieve due to the longer exposure times (up to 100 ms) required to integrate the fluorescence signal of our samples, i.e., we were not able to use the maximum camera speed.

In spite of the above, the proposed multicolor fluorescence imaging system was proven fast enough to track the dynamics of inner microstructures of a complex biological system such as *N. crassa* hyphae. We, therefore, anticipate that synchronizing the excitation lasers with a more sensitive single camera will allow the acquisition of dynamic multicolor 3-D images.

## 5 Conclusions

This work presents a multicolor fluorescence imaging system based on an SPIM configuration using three synchronized cw-lasers and a single-camera detection. Multicolor images of living hyphae of *N. crassa* expressing triple fluorescent markers were acquired with an acquisition rate up to 9 frames per s (three sequential frames for each laser). This is achieved without simultaneous laser excitation, and thereby, avoiding any bleed-through effects. This system is suitable for developmental biology studies and represents an alternative cost-effective approach to perform multicolor fluorescence imaging. This work has numerous applications in cell biology including fungal sexual development and host–pathogen interactions.
